# Obstetric Correlates of Maternal Falls in Southern Nigeria

**DOI:** 10.1155/2019/9716919

**Published:** 2019-07-25

**Authors:** Jacob Olumuyiwa Awoleke, Babatunde Ajayi Olofinbiyi, Adeola Olabisi Awoleke, Adefunke Christianah Omoyajowo

**Affiliations:** ^1^Department of Obstetrics and Gynaecology, Ekiti State University, Ado–Ekiti, Nigeria; ^2^Ekiti State University Teaching Hospital School of Nursing, Ado–Ekiti, Nigeria; ^3^Department of Mathematics and Statistics, Bowen University, Iwo, Nigeria

## Abstract

Falls during pregnancy can be associated with serious obstetric complications. Apart from sparse data highlighting traumatic outcomes, there are no studies identifying the obstetric correlates of maternal falls in Nigeria. A cross-sectional cohort survey of 1,175 women in five public health facilities in Ado–Ekiti was conducted to address this need. Fall rate was 25%; mothers who fell during pregnancy were significantly older, of higher parity, and with unintended/unwanted pregnancies than those who did not fall. Most of the reported falls occurred in the third trimester, with about 10% of the women falling at least thrice during the course of the pregnancy. More than half of the reported falls occurred while engaging in household chores and carrying child/object with compromised visibility of the feet and floor. Uterine contractions/abdominal pain was the commonest; 29 (76.3%), obstetric event attributed to the falls. Antepartum haemorrhage, 4 (10.5%), and ruptured membranes, 2 (5.3%), also occurred after falls, although it was rare and occurred with the same frequency as in the general population. Maternal age ≥ 30 years (odds ratio: 1.36; 95% C.I. 1.03 – 1.80, p = 0.031), multiparity (odds ratio: 1.54; 95% C.I. 1.15 – 2.07, p = 0.004), unintended pregnancy (odds ratio: 1.48; 95% C.I. 1.02 – 2.15, p = 0.037), and delivery age ≤ 40 weeks (odds ratio: 1.71; 95% C.I. 1.07 – 2.75, p = 0.026) were found to be independent risk factors for falls during pregnancy. Fall awareness campaigns and fall-preventing safety tips are advocated in women's clinics. Improving contraceptive uptake will reduce unintended pregnancies and the risk of pregnancy-related fall/injuries.

## 1. Introduction

Maternal falls have become an issue of public health importance, since studies have shown that at least one out of four women fall during pregnancy, with documented obstetric, perinatal, and orthopaedic complications [1–3]. Also, comparable fall rates have been noted between the elderly (about 27%) in the community and gravid women (about 26%) [4]. The increased risk of maternal falls has been linked to the anatomical and physiological changes during pregnancy, associated with factors such as altered biomechanics, displaced centre of gravity, and changes in joint kinetics. Thus, the more advanced the pregnancy is, the greater the anatomical changes, the more difficult it is to ambulate with ease, the poorer the visibility of the toes/floor, and the greater the likelihood of falling [5–9]. Although fatalities have been rarely documented from a fall in pregnancy, maternal traumatic morbidities that have resulted in unscheduled visits to the emergency departments, increased medical bills, and adverse perinatal outcomes have occurred [10–14].

Despite the fact that falls are the most common mechanism of injury during pregnancy [15], its prevalence, predictors, and obstetric correlates remain largely unknown in southern Nigeria. This knowledge gap has resulted in a neglect of awareness campaigns and a failure to develop locality-specific obstetric falls-risk assessment and preventive guidelines. This study has been designed to address this knowledge gap and serve as an exploratory survey of an emerging subject in Nigeria.

## 2. Method

The index study, a multifacility, cross-sectional cohort survey of women who had recently given birth until 9 months after delivery, was conducted in the postnatal wards, postnatal clinics, immunization units, and birth registration departments of public health facilities in Ado–Ekiti, the capital city of Ekiti State in southern Nigeria, between February and March, 2019. Ado–Ekiti has a population of 308,621 who are mostly Yoruba-speaking Christians and facilities providing maternal and child health services across the different tiers of healthcare [16]. According to the National Demographic and Health Survey 2013, Ekiti State has one of the highest percentages of officially registered births and immunization coverage in southern Nigeria [17]. These activities serve as a rallying point for postpartum women from across all social strata in Ekiti State.

Out of the thirteen (13) administrative wards in Ado–Ekiti Local Government Area, five wards were chosen by stratified sampling. One central public health facility was randomly selected in each of the five wards for the survey. These government-owned health institutions receive clients from the capital, other towns in Ekiti State, and from neighbouring Ondo, Kogi, and Osun states. Eligible participants were healthy women from second day postpartum to nine months post-delivery, who consented to participate in the survey. After the routine health instructions have been given, the purpose of the survey was explained, and semi-structured questionnaires were handed out to the consenting mothers. They were also given the option of opting out of the survey without repercussions.

We created a questionnaire based on other injury-evaluation instruments [1, 15, 18] for data collection. Women who could not read or write in English were interviewed by trained assistants who communicated the questions to them in Yoruba. The questionnaire was pretested in one of the facilities used in the survey to ascertain clarity and eliminate ambiguities. Information requested included age, area of residence, occupation, husband's occupation, monthly family income, marital status, parity, level of education and religion; the use of alcoholic beverages during the pregnancy and whether the pregnancy was wanted or not (unintended). The socioeconomic class was calculated after the model by Olusanya et al., with I being the highest and V the least [19]. Also, the women were asked about the number of falls, the gestational age of the latest fall (if they fell more than once) in months, the surrounding circumstances, obstetric complications attributable to the fall, and whether baby cried well at birth. The gestational age at delivery was obtained in weeks. However, for those who were not sure, it was calculated by extrapolating from the time interval between birth and their expected dates of delivery. Birthweights were confirmed from the immunization records of the babies when the mothers were unsure. To ensure robustness, 235 questionnaires were distributed in each facility for data collection. The self-reported questionnaires were then retrieved by the trained assistants. Where missing values occurred, the mean or modal values (whichever was appropriate) were imputed.

The survey was approved by the Ethics and Research Committee of the Ekiti State University Teaching Hospital, Ado–Ekiti, and the National Primary Health Care Development Agency, Ekiti State.

The retrieved data was coded into and analysed using the Statistical Software for the Social Sciences (SPSS) package version 20. Results were presented as percentages. Chi-square test was used for categorical variables; variables with p value < 0.05 were included in multivariate logistic regression analyses to identify the independent risk factors for maternal falls. The results were expressed as odds ratio at 95% confidence interval (C. I.), with level of significance set at p < 0.05.

## 3. Results

A total of 1,175 questionnaires were distributed in all the five public facilities. Of these, four (0.3%) were filled inadvertently by grannies who accompanied their daughters to the clinics, while 61 (5.2%) respondents opted out of the survey after initially consenting. Data analysis and result presentation were based on the 1,110 questionnaires that were correctly filled and returned. Two hundred and seventy-eight (25%) respondents reported at least a fall during pregnancy.

The demographic characteristics and obstetric correlates are as shown in Table 1. The mean age of the women was 30.26±4.93 years, with a range of 17–49 years. The mean parity was 2.1±1.2 (range: 1–9). The prevalence of falls during pregnancy was significantly increased in women who were at least 30 years old (62.9% versus 37.1%, p = 0.030), with unintended/unwanted pregnancies (31.8% versus 24%, p = 0.037), and who had not exceeded their expected delivery date (89.6% versus 10.4%, p = 0.025) compared with nonfallers. Also, multiparous women were at significantly increased risk of falling during pregnancy (70.9% versus 29.1%, p = 0.004). There was no significant association with place of residence, marital status, birthweight, perinatal outcome, and sex of the baby.

The number and timing of falls, causative factors and obstetric complications from the falls are displayed in Table 2. Most of the reported falls 154 (55.4%) occurred in the third trimester, with 27 (9.7%) of the women falling at least thrice during the course of the pregnancy. The commonest incidences of falls reported occurred while engaging in household chores 104 (37.4%), and carrying child/object with compromised visibility of the feet and floor 69 (24.8%). High-heeled shoes and footwears without adequate grip/backstraps were responsible for one-fifth (21.2%) of the falls. Nine (3.2%) of the respondents fell because they were shoved/struck mistakenly or deliberately, including during intimate partner violence. Of the 278 women who fell during pregnancy in this study, 38 (13.7%) had fall-related complications. This number also represents 3.4% of all the respondents. Uterine contractions/abdominal pain was the commonest 29 (76.3%) obstetric event attributed to the falls. Antepartum haemorrhage and ruptured membranes occurred in 4 (10.5%) and 2 (5.3%) respondents, respectively, after a fall.

From Table 3, multiple falls during pregnancy was predicted by multiparity (81.5% versus 18.5%, p = 0.034). Also, women who had three or more falls were significantly more likely to deliver babies weighing less than 4,000 grammes (70.4% versus 29.6%, p = 0.041). Other obstetric and demographic variables studied had no significant relationship with the number of falls.

Results of the multivariate logistic regression analysis of the variables significantly associated with maternal falls are shown in Table 4. Maternal age ≥ 30 years (odds ratio: 1.36; 95% C. I. 1.03 – 1.80, p = 0.031), multiparity (odds ratio: 1.54; 95% C. I. 1.15 – 2.07, p = 0.004), unintended pregnancy (odds ratio: 1.48; 95% C. I. 1.02 – 2.15, p = 0.037), and delivery age ≤ 40 weeks (odds ratio: 1.71; 95% C. I. 1.07 – 2.75, p = 0.026) were found to be independent risk factors for falls during pregnancy.

## 4. Discussion

The analyses showed that one-quarter (25%) of the respondents had a fall during pregnancy. This figure tallies with the reported prevalence from other studies [1, 20]. The high incidence of falls becomes even more worrisome because about 10% of these women fell at least thrice during pregnancy, with associated complications. This could worsen the existing grim statistics on maternal and perinatal morbidity in Nigeria.

Our study revealed that women 30 years and above were significantly more likely to fall during pregnancy, controverting studies that found higher incidence of falls in younger women because they are more active [1, 21]. The observed variance could be due to geographical/racial differences, sample sizes of the studies, and cultural diversity of the population studied. Also, it appears that increased activity was not the only factor responsible for falls in the other studies, because McCrory et al. [22] found that sedentary pregnant women were more likely to experience a fall. Factors that increase the fall risk in older nonpregnant women, such as reduced perception of their risk of falling, and postural instability [23, 24], may also be playing out in these older pregnant women. Since dynamic balance is altered in pregnant fallers compared with nonfallers, and exercise participation during pregnancy is encouraged [25]. Exploring the option of guided exercises during pregnancy as a fall-prevention measure might be worth the effort.

Significantly, multiparous women were more likely to fall during pregnancy from our survey. Although another study from southeast Nigeria concluded otherwise [21], the sample size was much smaller. Multiparous women were more likely to be involved in domestic duties as they care for their large families. These can increase the proneness to and the number of falls in them. In southwestern Nigerian families, the more the children, the more the domestic challenges, chores. and responsibilities. We believe that as these duties increase for the mothers during pregnancy, so also does the tendency to tripping and falling. This is supported by the finding that the commonest scenarios for the falls in this study were while doing household chores and carrying child/object. Displaying and distributing pamphlets with safety rules such as unhurried carefulness while carrying a child/object, use of flat, firm-gripping footwears, avoiding slippery floors, and using the rails while climbing stairs, should be encouraged in prenatal classes.

Unintended pregnancies predicted the risk of falling from our study. Although the role of pregnancy intention/desire in injury risk has not been extensively explored, unintended pregnancies have been linked with poor health-seeking behaviours (such as continued alcohol use in pregnancy) and adverse pregnancy outcomes (including preterm deliveries and low-birth weight babies) [26, 27]. This association is obviously not straightforward and could be casual, rather than causal. Psychologically, self-care may be neglected when a pregnancy is unwanted, leading to an increased risk of injury to both maternal and fetal health. The effects of unintended pregnancy can be modified through preconceptional education and family planning. Larger studies are needed to identify the possible contribution of unintended pregnancy to the prevalence of maternal falls.

Most of the falls reportedly occurred in the third trimester, with about 10% of the women falling at least thrice. Thus, as pregnancy advances, the shift in the centre of gravity occasioned by the protruding abdomen results in a decrease in postural stability [20, 22]. The associated increase in postural sway in the second and third trimesters adds to the instability and worsen the fall risks [20, 28].

Significant obstetric complications were observed after falls in this survey. Adverse pregnancy outcomes have been found in supposedly minor injuries [29]. For example, of the 278 women with falls, four (1.4%) had antepartum haemorrhage. This is similar to its non-fall-related prevalence in the general population [30]. These pregnancy complications could result in earlier deliveries in other to preserve maternal and/or fetal health. The fact that most pregnant fallers from this study did not exceed their due dates supports this concern. Also, women who fell three or more times had a higher percentage of babies with birth weight greater than 4 kg when compared with non-fallers. Maybe the birth weight here is more related to multiparity and age of the women who fell more times, rather than to prematurity. Identifying these obstetric correlates will inform the development of locality-specific obstetric falls-risk assessment and prevention guidelines.

This survey may be limited by participants' recall bias and underreporting. However, these potential confounders were reduced by its adequate sample size calculated within a narrow error margin, and regression analyses to confirm statistical significance.

## 5. Conclusion

Falls occur commonly among our pregnant population, most events occurring in late trimester with associated obstetric complications. The risks are greater in older, multiparous women, especially those with unintended pregnancy. Scaling up awareness campaigns and educating the women about fall-prevention safety tips during pregnancy are encouraged. Preconceptional education and increased contraceptive uptake in Nigeria can reduce the burden of, or contribution from, falls in women with unintended pregnancy. Developing locality-specific obstetric falls-risk assessment and prevention guidelines will be a step in the right direction.

## Figures and Tables

**Table 1:** The demographic and obstetric characteristics of the respondents.

Variables	Categories	No Fall	Fall	*p* value
		n (%)	n (%)	
*Age group (years)*	< 30	370 (44.5)	103 (37.1)	0.030*∗*
	≥ 30	462 (55.5)	175 (62.9)	

*Residence *	Rural	306 (36.8)	111 (39.9)	0.348
	Urban	526 (63.2)	167 (60.1)	

*Marital Status*	Single	35 (4.2)	5 (1.8)	0.062
	Married	797 (95.8)	273 (98.2)	

*Educational level*	No formal	7 (0.8)	2 (0.7)	0.968
	Primary	24 (2.9)	7 (2.5)	
	Secondary	235 (28.2)	76 (27.3)	
	Tertiary	566 (68)	193 (69.4)	

*Monthly income*	< $150	333 (40)	120 (43.2)	0.075
	$150 – $199	315 (37.9)	81 (29.1)	
	$200 – $249	91 (10.9)	42 (15.1)	
	$250 – $299	38 (4.6)	13 (4.7)	
	≥ $300	55 (6.6)	22 (7.9)	

*Social class*	I	124 (14.9)	47 (16.9)	0.837
	II	233 (28)	70 (25.2)	
	III	268 (32.2)	89 (32)	
	IV	193 (23.2)	66 (23.7)	
	V	14 (1.7)	6 (2.2)	

*Pregnancy wanted*	Yes	727 (76)	229 (24)	0.037*∗*
	No	105 (68.2)	49 (31.8)	

*Alcohol use*	No	775 (93.1)	264 (95)	0.284
	Yes	57 (6.9)	14 (5)	

*Age at delivery (weeks)*	≤ 40	779 (93.6)	249 (89.6)	0.025*∗*
	Above 40	53 (6.4)	29 (10.4)	

*Birthweight (grammes)*	Mean ± SD	3319±580	3290±574	0.480

*Birthweight *	< 4,000	722 (86.8)	240 (86.3)	0.849
	≥ 4,000	110 (13.2)	38 (13.7)	

*Sex of the baby*	Male	411 (49.4)	136 (48.9)	0.890
	Female	421 (50.6)	142 (51.1)	

*Baby cried at birth*	Yes	818 (98.3)	271 (97.5)	0.376
	No	14 (1.7)	7 (2.5)	

*Parity distribution *	1 (primiparous)	323 (38.8)	81 (29.1)	0.004*∗*
	≥ 2 (multiparous)	509 (61.2)	197 (70.9)	

Chi-square test was employed; *∗*significant at *p *< 0.05.

**Table 2:** Overview of the falls, n = 278.

	n (%)
*Gestational age at fall (in months)*	
≤ 3	18 (6.5)
4 – 6	106 (38.1)
≥ 7	154 (55.4)

*Number of falls during the pregnancy (Range: 1 - 6)*	
1 – 2	251 (90.3)
≥ 3	27 (9.7)

*Causative factors for the falls * **∗**	
During household chores	104 (37.4)
Carrying child/object with poor visibility of the toes/floor	69 (24.8)
Inappropriate shoes	59 (21.2)
Slipped on floor or in the bathroom	48 (17.3)
Hurrying	48 (17.3)
Climbing stairs	20 (7.2)
Shoved or struck by someone else	9 (3.2)

*Obstetric complications attributable to the fall∗(n = 38)*	
Uterine contractions	29 (76.3)
Reduced or absent fetal movement	5 (13.2)
Ruptured membranes	2 (5.3)
Antepartum haemorrhage	4 (10.5)

**∗**Total > 100% due to multiple responses.

**Table 3:** Relationship between the demographic and obstetric characteristics of the women and the number of falls.

Variables	Categories	Number of falls in pregnancy			*p* value
		0	1 – 2	≥ 3	
		n (%)	n (%)	n (%)	
*Age group (years)*	< 30	367 (44.1)	97 (38.6)	9 (33.3)	0.189
	≥ 30	465 (55.9)	154 (61.4)	18 (66.7)	

*Residence *	Rural	305 (36.7)	102 (40.6)	10 (37)	
	Urban	527 (63.3)	149 (59.4)	17 (63)	

*Marital Status*	Single	35 (4.2)	4 (1.6)	1 (3.7)	0.150
	Married	797 (95.8)	247 (98.4)	26 (96.3)	

*Educational level*	No formal	7 (0.8)	2 (0.8)	0 (0)	0.770
	Primary	25 (3)	5 (2)	1 (3.7)	
	Secondary	230 (27.6)	70 (27.9)	11 (40.7)	
	Tertiary	570 (68.5)	174 (69.3)	15 (55.6)	

*Monthly income*	< $150	333 (40)	109 (43.4)	11 (40.7)	0.597
	$150 – $199	311 (37.4)	76 (30.3)	9 (33.3)	
	$200 – $249	91 (10.9)	38 (15.1)	4 (14.8)	
	$250 – $299	39 (4.7)	11 (4.4)	1 (3.7)	
	≥ $300	58 (7)	17 (6.8)	2 (7.4)	

*Social class*	I	125 (15)	43 (17.1)	3 (11.1)	0.537
	II	234 (28.1)	66 (26.3)	3 (11.1)	
	III	269 (32.2)	78 (31.1)	10 (37)	
	IV	190 (22.8)	59 (23.5)	10 (37)	
	V	14 (1.7)	5 (2)	1 (3.7)	

*Pregnancy wanted*	Yes	726 (87.3)	209 (83.3)	21 (77.8)	0.123
	No	106 (12.7)	42 (16.7)	6 (22.2)	

*Alcohol use*	No	776 (93.3)	238 (94.8)	25 (92.6)	0.663
	Yes	56 (6.7)	13 (5.2)	2 (7.4)	

*Age at delivery (weeks)*	≤ 40	779 (93.6)	224 (89.2)	25 (92.6)	0.066
	Above 40	53 (6.4)	27 (10.8)	2 (7.4)	

*Birthweight *	< 4,000	725 (87.1)	218 (86.9)	19 (70.4)	0.041*∗*
	≥ 4,000	107 (12.9)	33 (13.1)	8 (29.6)	

*Sex of the baby*	Male	409 (49.2)	124 (49.4)	14 (51.9)	0.962
	Female	423 (50.8)	127 (50.6)	13 (48.1)	

*Baby cried at birth*	Yes	818 (98.3)	245 (97.6)	26 (96.3)	0.604
	No	14 (1.7)	6 (2.4)	1 (3.7)	

*Parity distribution *	1 (primiparous)	318 (38.2)	81 (32.3)	5 (18.5)	0.034*∗*
	≥ 2 (multiparous)	514 (61.8)	170 (67.7)	22 (81.5)	

Chi-square test was employed; *∗*significant at *p *< 0.05.

**Table 4:** Multiple logistic regression of risk factors associated with maternal falls.

Characteristics	Odds ratio	95% C. I.	*p* value	Reference
Age ≥ 30 years	1.361	1.03 – 1.80	0.031*∗*	Age < 30 years
Multiparity	1.543	1.15 – 2.07	0.004*∗*	Primiparity
Unwanted pregnancy	1.482	1.02 – 2.15	0.037*∗*	Wanted pregnancy
Delivery age ≤ 40 weeks	1.712	1.07 – 2.75	0.026*∗*	Delivery age > 40 weeks

*∗*Significant at *p *< 0.05.

## Data Availability

The data used to support the findings of this study are available from the corresponding author upon request.
